# Genome-Wide Transcriptome Analysis of a Virulent sRNA, Trans217, in *Xanthomonas oryzae* pv. *oryzae* (*Xoo*), the Causative Agent of Rice Bacterial Blight

**DOI:** 10.3390/microorganisms12081684

**Published:** 2024-08-16

**Authors:** Yiqun Hu, Jianjian Zhang, Aifang Zhang

**Affiliations:** 1 Institute of Plant Protection and Agro-Product Safety, Anhui Academy of Agricultural Sciences, Hefei 230031, China; huyiqun@aaas.org.cn; 2 Anhui Province Key Laboratory of Pesticide Resistance Management on Grain and Vegetable Pests, Hefei 230031, China; 3 Department of science research University of Science and Technology of China, Hefei 230026, China; zhangjianjian@ustc.edu.cn

**Keywords:** *Xanthomonas oryzae* pv. *oryzae*, sRNA trans217, transcriptome, gene differential expression, oxidative stress

## Abstract

Small non-coding RNAs (sRNAs) act as post-transcriptional regulators to participate in many cellular processes. Among these, sRNA trans217 has been identified as a key virulent factor associated with pathogenicity in rice, triggering hypersensitive reactions in non-host tobacco and facilitating the secretion of the PthXo1 effector in *Xanthomonas oryzae* pv. *oryzae* (*Xoo*) strain PXO99^A^. Elucidating potential targets and downstream regulatory genes is crucial for understanding cellular networks governing pathogenicity and plant resistance. To explore the targets regulated by sRNA trans217, transcriptome sequencing was carried out to assess differential expression genes (DEGs) between the wild-type strain PXO99^A^ and a mutant lacking the sRNA fragment under both virulence-inducing or normal growth conditions. DEG analysis revealed that sRNA trans217 was responsible for diverse functions, such as type III secretion system (T3SS), glutamate synthase activity, and oxidative stress response. Three genes were selected for further investigation due to their significant differential expression and biological relevance. Deletion of *PXO_RS08490* attenuated the pathogenicity of *Xoo* in rice and reduced the tolerance level of PXO99^A^ to hydrogen peroxide. These findings suggest a regulatory role of sRNA trans217 in modulating bacterial virulence through multiple gene targets, either directly or indirectly.

## 1. Introduction

Small regulatory RNAs (sRNAs) serve as important post-transcriptional modulators for bacterial adaptation to environmental changes, antibiotic resistance, and bacterial virulence [1,2,3,4,5]. Although sRNAs were identified many decades ago, detailed mechanisms have been elucidated in only a few bacterial genera, such as *Escherichia coli* and *Salmonella* [6,7,8,9]. Extensive research on several Gram-negative species has established them as excellent model organisms for sRNA biology [10,11,12]. In *E. coli*, the sRNA RyhB has been found to significantly impact various biological processes. It interacts with ferric uptake regulators to control colibactin production, regulate *clbA* gene transcription, and manage cellular iron homeostasis [13,14,15]. RyhB also mediates resistance to multiple antibiotics and stress conditions by reducing cellular metabolism [16]. Another sRNA, InvS, coordinates bacterial invasion by regulating the levels of FimZ (a repressor of invasion-associated gene expression) and PrgH (a component of the type III secretion system) [17]. The trans-encoded sRNA SgrS negatively regulates the expression of several mRNAs under phosphoglucose stress [18]. Specifically, it inhibits the translation of *ptsG* mRNA by base pairing between a short sequence in the 3′ UTR and the Shine–Dalgarno sequence [19].

In recent years, the role of sRNAs in gene regulation has attracted considerable attention across a diverse array of species, including Gram-positive bacteria [20,21,22]. However, only a few studies have been conducted on plant-pathogenic bacteria, also in *Xanthomonas* species [23]. The sRNA Xonc3711 of *Xoc* binds to the gene coding region of *Xoc_3982*, which encodes a DNA-binding protein. This interaction negatively regulates the generation of *Xoc_3982* mRNA, suppresses the expression of flagella-associated genes and biofilm formation, affects the antioxidant defenses of bacteria, and thus regulates bacterial toxicity [24]. A specific sRNA, RsmU, identified through genome-wide screening in *X. campestris* pv.*campestris* (*Xcc)*, negatively regulates bacterial virulence, anaphylaxis, and cell motility. In vitro electrophoretic mobility transfer assays and in vivo co-immunoprecipitation analyses revealed that RsmU interacts with the global post-transcriptional regulator RsmA. Northern blotting analysis identified two isoforms of RsmU processed from the 3′-UTR of *XC1332* mRNA [25]. Genome-wide transcriptome analysis in *X. campestris* pv.*vesicatoria* (*Xcv*) identified twenty-four sRNAs, of which eight are regulated by HrpG and HrpX. Both sX12 and sX13 contribute to bacterial virulence. sX12 facilitates bacterial adaptation to the environment by influencing the interaction between *Xanthomonas* and its host, rapeseed. sX13 promotes HrpX mRNA accumulation, and its deletion affects *Xcv* virulence and T3SS-related gene expression. sX13 acts upstream of HrpG but does not affect HrpG mRNA levels. Using a GFP reporter system, it was shown that sX13-mediated inhibition of protein synthesis relies on the binding of C-rich sequences to target mRNA. Although the chaperone protein Hfq is not essential for sX13 activity, sX13 negatively regulates Hfq expression levels [26]. An RNomics approach identified four sRNAs in *Xcc*, namely, Xcc1, Xcc2, Xcc3, and Xcc4. Xcc1 can function as an exchange sRNA for integron-encoded transposons and plasmid transfer and is positively modulated by the HrpX and HrpG factors [27].

*Xanthomonas oryzae* pv. *oryzae* (*Xoo*) is a plant-pathogenic bacterium that induces bacterial blight, resulting in a significant loss of rice quality and yield [28,29]. The trans217 sRNA was identified and characterized as a virulence-associated sRNA through high-throughput sequencing and genetic and functional studies in the *Xoo* strain PXO99^A^ [30]. The importance of the trans217 sRNA has been demonstrated in *Xoo* pathogenicity in rice, the hypersensitive response (HR) in tobacco, and the expression of T3SS [30]. It is evident that sRNA trans217 is part of a complex regulatory network with various functions, necessitating further investigation to elucidate its precise regulatory mechanisms.

This study is a follow-up on sRNA trans217. The regulatory network of the trans217 sRNA was analyzed by comparing the transcriptomes of wild-type (WT) and mutant strains lacking the trans217 sRNA. Three differentially expressed genes were selected, and corresponding gene deletion strains were generated. Their regulatory functions were determined through pathogenicity and phenotypic assays. The transcriptome data provide extensive details about downstream regulators of the trans217 sRNA involved in virulence.

## 2. Materials and Methods

### 2.1. Xoo RNA Sequencing

The transcriptome sequencing process began with total RNA extraction from each sample and ribosomal RNA elimination using the Ribo-Zero kit. The RNA was fragmented into short sequences, and single-strand cDNA was synthesized using random hexamer primers. Double-strand cDNA synthesis followed, with dUTP substituted for dTTP. Different adaptors were attached, and the strand containing dUTP was digested using the UNG enzyme method, leaving only one strand of cDNA. This cDNA strand was purified, end-repaired, and had A-tails added. Sequencing adaptors were then connected, fragment size was chosen, and PCR amplification was conducted. The constructed libraries were validated using the Agilent 2100 Bioanalyzer, followed by sequencing with an Illumina sequencer.

High-throughput sequencing generated raw reads. fastp (0.20.1) software was used to remove adaptors and N bases for obtaining clean reads. These clean reads were mapped against the reference genome using Rockhopper2 (2.0.3) software to determine location information and sample-specific sequence characteristics. Gene expression abundance and RPKM values were computed based on the counts of reads mapped to genomic regions or gene exons.

### 2.2. Enrichment Analysis of the DEGs

DESeq2 was utilized to normalize the gene counts for each sample (with BaseMean values estimating expression levels), calculate fold change differences, and test the significance of these differences using a NB test. Differentially expressed protein-coding genes were then screened on the basis of fold change differences and statistical significance. The transcripts with a *p*-value less than 0.05 and difference of multiples of more than two were selected out as DEGs. After identifying the DEGs, we analyzed the enriched GO terms and KEGG pathways.

GO and KEGG were employed for pathway analysis of differentially expressed protein-coding genes to identify their potential pathway associations. The significance of DEG enrichment across pathway entries was evaluated using a hypergeometric distribution test. Lower *p*-values (near zone) indicated a stronger relevance of the DEGs to the specific pathways.

### 2.3. Verification of DEGs between the XOM2 and PSA Medium

Gene expression levels were quantified as previously described [31]. To validate the RNA-Seq data, selected DEGs were quantified by RT-qPCR using specific primers (Additional file 1: Table S1). The RT-qPCR assay was performed in a 25 µL mixture containing 1 µL of 1:10 diluted first-strand cDNA, 2.5 µM of each primer, and 1 × SYBR Premix Ex Taq (TaKaRa). Each reaction was conducted in triplicate with no template controls where cDNA was omitted. The *16S rRNA* gene, which is constitutively expressed, served as the reference. The relative expression levels of the DEGs were computed in accordance with the Ct-value ratio of each DEG compared to *16S rRNA* [32].

### 2.4. Gene-Directed Mutagenesis and Complementation Assays

To construct gene deletion mutants, the flanking sequences of upstream/downstream genes were amplified from PXO99^A^ using sequence-specific primer pairs (Additional file 1: Table S1). Following PCR, the products were treated with specific restriction enzymes and cloned into the suicide vector pK18mab*SacB* (Additional file 1: Table S1). These recombinant vectors were introduced into competent PXO99^A^ cells via electroporation. The transformed cells were plated on NA medium lacking sugar and containing 50 μg/mL kanamycin and incubated at 28 °C for three days. Single colonies were subsequently verified through PCR. Positive clones were further tested by spreading on NA medium with 10% sucrose, incubating for 3 days, and conducting PCR assays to confirm gene deletion mutants. For complementation, sRNA PCR fragments were ligated into the expression vector pHM1 using specific restriction enzymes (Additional file 1: Table S1). Competent cells of the corresponding deletion mutant were then electro-transformed with the recombinant vector, and transformants were verified using the same method as for the mutants.

### 2.5. Virulence Assessment

The plants were grown in a plant growth chamber under 26 °C, 85% humidity, and a 14 h light cycle at 250 μE/m^2^/s. In the plant inoculation assay, one-month-old rice plant leaves were clipped with sterile scissors and immersed in bacterial cultures (OD600 ≈ 0.5) approximately 2 cm from the leaf edge. Each bacterial strain was tested on 5 plants, with 3 leaves per plant. Lesion length was measured two weeks post-inoculation, and the mean lesion length was computed. To assess bacterial populations in leaf tissues, the whole leaves were cut into segments, followed by sterilization with 75% (*v*/*v*) ethanol and homogenization in sterile water. The bacteria were then isolated by culturing the homogenates on NA medium. Bacterial counts were determined as colony-forming units (CFUs). Bacterial blight severity (lesion length) and bacterial populations in leaf tissues were used to assess the virulence of diverse *Xoo* strains.

### 2.6. Bacterial Growth Curve Determination

Fresh *Xoo* cultures (OD600 ≈ 1.0, 10 µL) were transferred into a new bottle containing 20 mL of medium and incubated at 28 °C with shaking. The abundance of bacteria was determined at 4 h intervals over a period of 20 to 40 h.

### 2.7. Oxidative Stress Assessment

For the oxidative stress assessment, bacterial cultures were adjusted to OD600 values of approximately 1 and 0.2. Then, 2 µL of each culture was plated onto NY agar plates (per liter: 15 g agar, 3 g polypeptone, 1 g yeast extract, pH 6.5). Hydrogen peroxide solutions at concentrations of 0, 0.1, 0.2, 0.3, and 0.4 mM were added to the plates. The plates were incubated at 28 °C for three days, and images were captured. The experiment was conducted in triplicate.

## 3. Results

### 3.1. Overview of Comparative Transcriptomic Analyses

**Figure 1:**
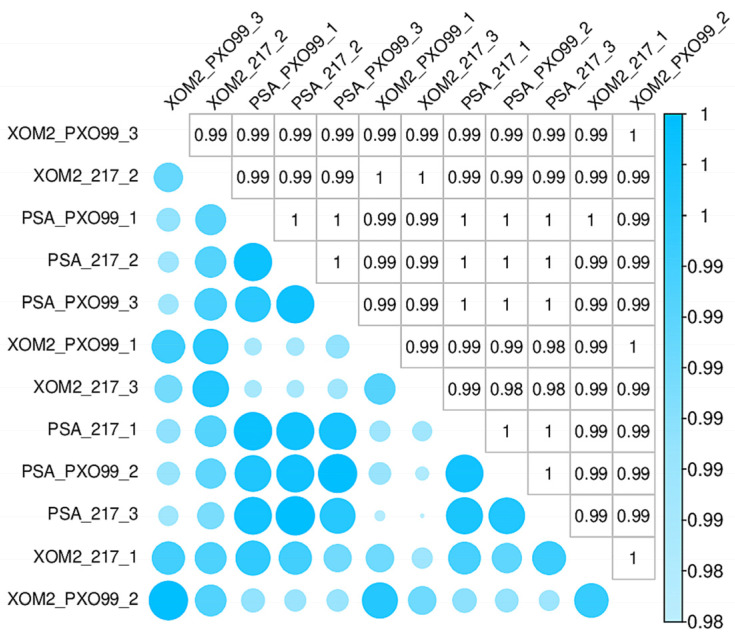
Heat map of correlation coefficients between the wild-type strain PXO99^A^ of *Xanthomonas oryzae* pv. *oryzae* (*Xoo*) and the mutant strain lacking the trans217 sRNA (Δtrans217). XOM2 (*X. oryzae* medium 2) was used for virulence induction and PSA (polypeptone sucrose agar) was used for standard growth. The horizontal axis shows the specimen identities, the vertical axis lists the corresponding specimen identities, and the colors denote the correlation coefficients.

The strains used for transcriptome sequencing were PXO99^A^ and Δtrans217. Bacterial RNA was collected after growth in *X. oryzae* recipe 2 (XOM2) and polypeptone sucrose agar (PSA) media for virulence induction and normal growth, respectively, with three biological replicates for each sample, totaling 12 samples. After assessing the quality of the RNA samples, libraries were constructed, and RNA-Seq was performed. Following the removal of splice junctions and low-quality reads, 29.02 G of high-quality reads were acquired. Each sample had an effective data volume ranging from 2.32 to 2.54 G, with Q30 base distribution between 93.68% and 94.71%, and a mean GC content of 60.77%. The reference genome was used for aligning the reads, with alignment and mapping rates ranging from 81.0% to 92.0% (Table 1). Correlation analyses of the three biological replicates in different groups yielded correlation coefficients greater than 0.9, indicating high reproducibility in the sequencing results. This high reproducibility ensures the reliability of subsequent evaluations of differentially expressed genes (Figure 1).

### 3.2. Validation of the Transcriptome Data with Quantitative Real-Time PCR

**Figure 2:**
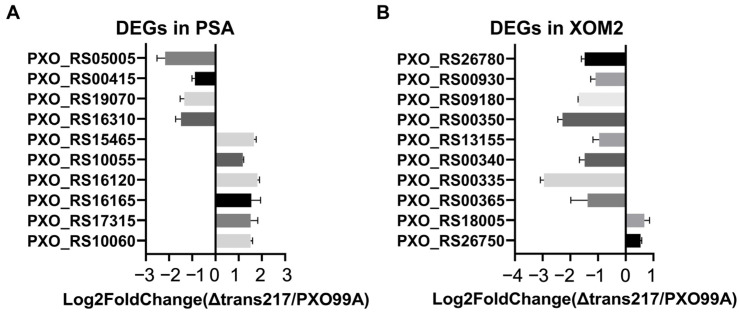
Verification of the transcriptome data by RT-qPCR. Expression levels of DEGs in *Xoo* PXO99^A^ and a mutant strain lacking sRNA 217 (Δtrans217) grown in a nutrient-rich polypeptone sucrose agar (PSA) medium (**A**) and nutrient-deficient XOM2 conditions (**B**).

Read counts were normalized, gene expression differences between sample groups were identified, and fold changes were calculated using DESeq2 with default parameter settings [32]. The negative binomial distribution (NB) test was employed to assess significance. Based on the above results, differentially expressed protein-coding genes were screened. To validate the robustness of the transcriptome data disclosed by the sequencing provider, RT-qPCR assay was employed to quantify gene expression. Twenty genes were randomly chosen from the transcriptome data of the PSA and XOM2 groups. The findings indicated that the expression levels of these 20 genes aligned with those acquired from transcriptome sequencing (Figure 2). Although gene expression levels varied, the trends of up-regulation and down-regulation were consistent.

### 3.3. Quantitative and Categorical Analysis of the DEGs

**Figure 3:**
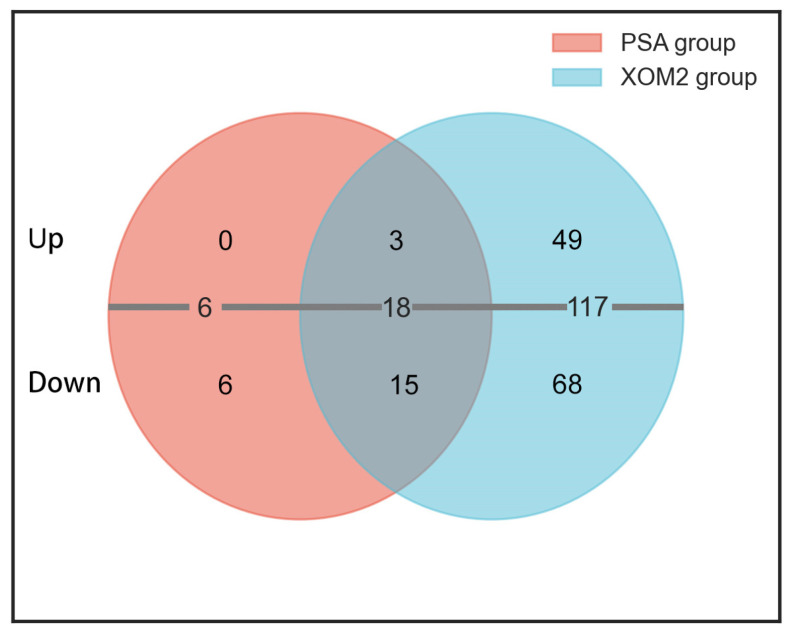
Venn diagram analysis illustrating the number of DEGs in *Xoo* cultured on the PSA and XOM2 media. RNA-Seq identified a total of 141 DEGs, categorized based on their up- or down-regulation and the growth media used, PSA or XOM2.

Twenty-four differentially expressed genes were identified in the PSA culture by comparing the transcriptomic data of wild-type PXO99^A^ and Δtrans217 strains according to the DEG screening criteria of FDR ≤ 0.01 and |log2FC| > 0.58. Among these genes, 3 were upregulated and 21 were downregulated. In the XOM2 culture, 135 DEGs were identified, with 52 upregulated and 83 downregulated. When |log2FC| > 1, 6 DEGs were found in the PSA group, all of which were upregulated. In contrast, the XOM2 group showed 33 DEGs, including 7 upregulated and 26 downregulated genes (Figure 3). Overall, there was a predominance of downregulated genes compared to upregulated genes.

**Figure 4:**
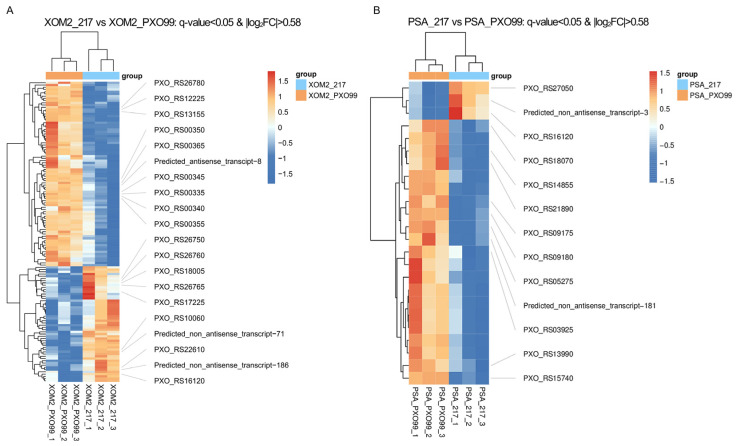
Heatmap analysis of DEGs. DEGs in the XOM2 (**A**) and PSA (**B**) groups. Red corresponds to high-expression genes. Blue corresponds to low-expression genes. The *X*-axis and *Y*-axis denote the sample name and gene name, respectively.

In addition, heatmap analysis provided an overview of the overall distribution of differentially expressed genes (Figure 4). The findings indicated that the number of genes induced by the XOM2 culture was notably higher than those differentially expressed under the PSA culture, with a significantly higher count of downregulated genes compared to upregulated genes.

### 3.4. Enrichment Analyses of DEGs

**Figure 5:**
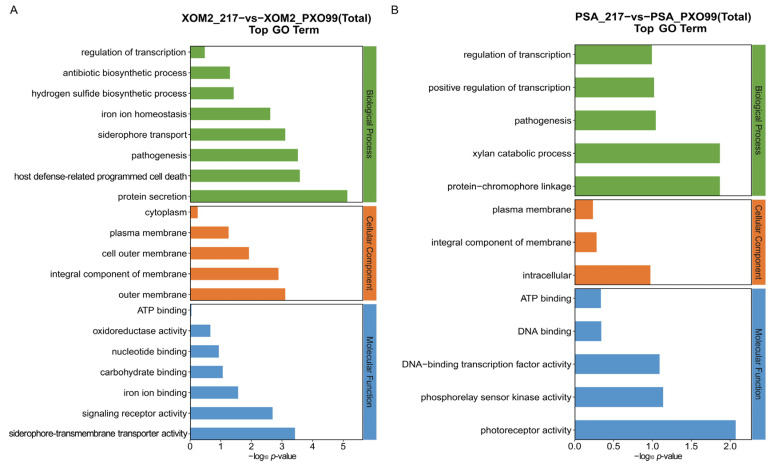
Summary of GO enrichment analysis. Horizontal and vertical axes indicate GO entry name and −log_10_ *p*-value, respectively. XOM2 (**A**) and PSA (**B**) groups were compared to PXO99^A^, respectively.

GO enrichment analysis was conducted to provide an initial overview of gene function. The top 30 most enriched GO terms, categorized into biological process, cellular component, and molecular function, are presented in the bar chart below (Figure 5). These GO terms were selected based on having more than two corresponding DEGs and are ranked by −log_10_ *p*-value. In the XOM2 group, GO enrichment indicated that the enriched biological processes were related to protein secretion, pathogenic bacteria, and host defense. This implies that trans217 sRNA is responsible for interactions between the pathogen and its host.

**Figure 6:**
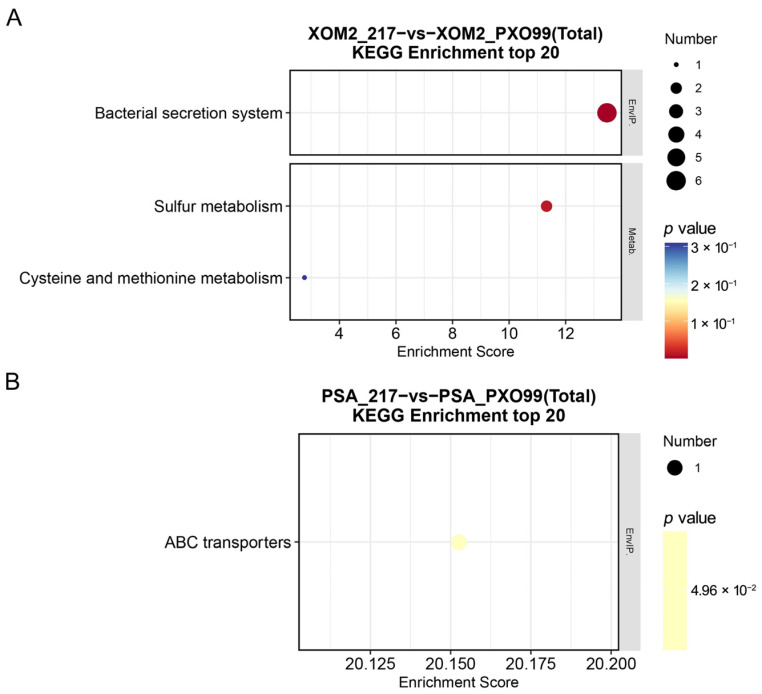
Summary of KEGG enrichment analysis. XOM2 (**A**) and PSA (**B**) groups were compared to wild-type strain PXO99^A^, respectively. Horizontal axis indicates enrichment scores. Entries with more bubbles contain higher number of differentially expressed protein-coding genes, and the colors of the bubbles range from blue to yellow to red. A lower enrichment *p*-value corresponds to greater significance.

Further analysis was conducted to investigate the involvement of DEGs in various pathways. For KEGG enrichment analysis, the top 20 pathway entries with more than two DEGs were selected and ranked from highest to lowest based on their −log_10_ *p*-values. The corresponding bubble diagram is depicted below (Figure 6). The analysis revealed that approximately half of the DEGs in the XOM2 group were associated with bacterial secretory system functions, whilst the remaining genes were involved in sulfur metabolism, cysteine, and methionine metabolism. In contrast, DEGs in the PSA group were linked to processes related to ABC transporters. The bacterial secretory system, particularly T3SS, is crucial for injecting effectors into host cells to facilitate virulent activities [33,34]. XOM2, characterized by nutrient limitation, can enhance the expression and activation of virulence-related genes. The KEGG data suggest that sRNAs play a role in bacterial virulence by modulating the bacterial secretion system.

### 3.5. PXO_RS08490 Contributes to Bacterial Virulence

**Figure 7:**
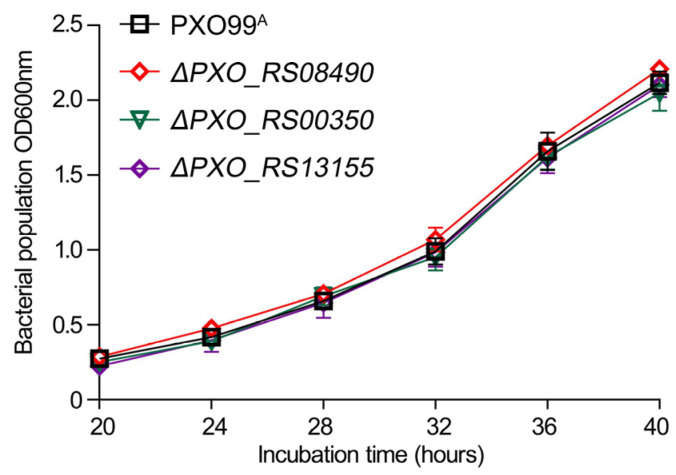
Measurement of the bacterial growth rate of *Xoo* strains. *Xoo* PXO99^A^ and deletion mutant strains were cultured on a nutrient medium. The OD600 values represent bacterial cell density. Data at each time point are presented as mean ± SD (*n* = 5).

**Figure 8:**
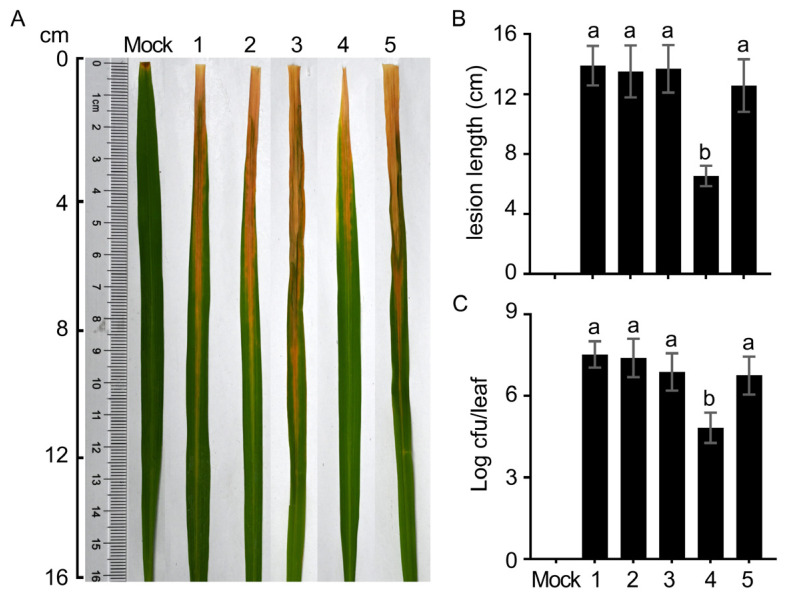
Virulence assessment of *Xoo* strains. Virulence assessments were conducted on the susceptible rice variety, Nipponbare. The tested strains were PXO99^A^ (1), Δ*PXO_RS13155* (2), Δ*PXO_RS00350* (3), Δ*PXO_RS08490* (4), and CΔ*PXO_RS08490* (5). (**A**) Images were captured on day 14 after inoculation using the leaf-clipping method to record bacterial blight symptoms on Nipponbare leaves. (**B**) Lesion length due to blight was measured (*n* = 10 leaves). (**C**) Bacterial populations within Nipponbare leaves were quantified 3 days following infiltration inoculations at the leaf center (*n* = 3 experimental replicates). Data are presented as mean ± SD. Different lowercase letters denote significant differences based on analysis of variance with Fisher’s least significant difference test (*p* < 0.01).

Transcriptome data provide guidance for subsequent molecular function studies, necessitating the alteration of specific functions to generate mutants and validate their roles in conjunction with experimental phenotypes. Three DEGs were selected from the transcriptome data to assess the effect of trans217 sRNA deletion on downstream genes. These included *PXO_RS08490* encoding superoxide dismutase (SOD), *PXO_RS00350* encoding T3SS cytoplasmic ring protein, and *PXO_RS13155* encoding glutamate synthase. A homologous recombination approach was employed to construct mutant strains carrying these gene deletions. Initially, bacterial growth rates were measured to confirm that the deletion of the three genes did not affect bacterial growth (Figure 7). Subsequently, the pathogenicity of *Xoo* in rice hosts was evaluated. Inoculation with wild-type PXO999^A^ resulted in typical symptoms of bacterial blight, characterized by yellow and white spots on the upper side of leaves that became wrinkled, with spot lengths exceeding 10 cm. Similar symptoms were observed for strains lacking *PXO_RS13155* and *PXO_RS00350* compared to PXO999^A^. However, Δ*PXO_RS08490* mutants exhibited significantly milder symptoms, with reduced lesion length and fewer colonizing bacteria compared to the wild type (Figure 8). Importantly, reconstitution of the *PXO_RS08490* gene in the Δ*PXO_RS08490* mutant strain restored lesion length to wild-type levels (Figure 8).

### 3.6. PXO_RS08490 Regulates Oxidative Stress

When plants are infected by pathogens, they trigger a sequence of defense responses, among which oxidate burst is one of the most rapid reactions. The process generates a large amount of reactive oxygen species [35], and the capability of pathogens to resist oxidative stress is crucial for the successful infection of plants.

The gene *PXO_RS08490* encodes a superoxide dismutase (SOD), a type of antioxidant enzyme critical for maintaining normal oxidative stress levels in bacteria. SODs can catalyze the disproportionation reaction of superoxide anion free radicals to produce H_2_O_2_ and O_2_, thereby neutralizing harmful free radicals generated during bacterial aerobic metabolism and protecting cells from oxidative damage. SODs play a critical role not only in balancing self-oxidation and anti-oxidation within bacteria but also in maintaining oxidative stress homeostasis during interactions between bacteria and their external environment. It was important to investigate whether deletion of the SOD gene would affect bacterial growth under conditions of exogenous H_2_O_2_. Therefore, we assessed oxidative stress in bacteria following exposure to varying concentrations of exogenous H_2_O_2_.

**Figure 9:**
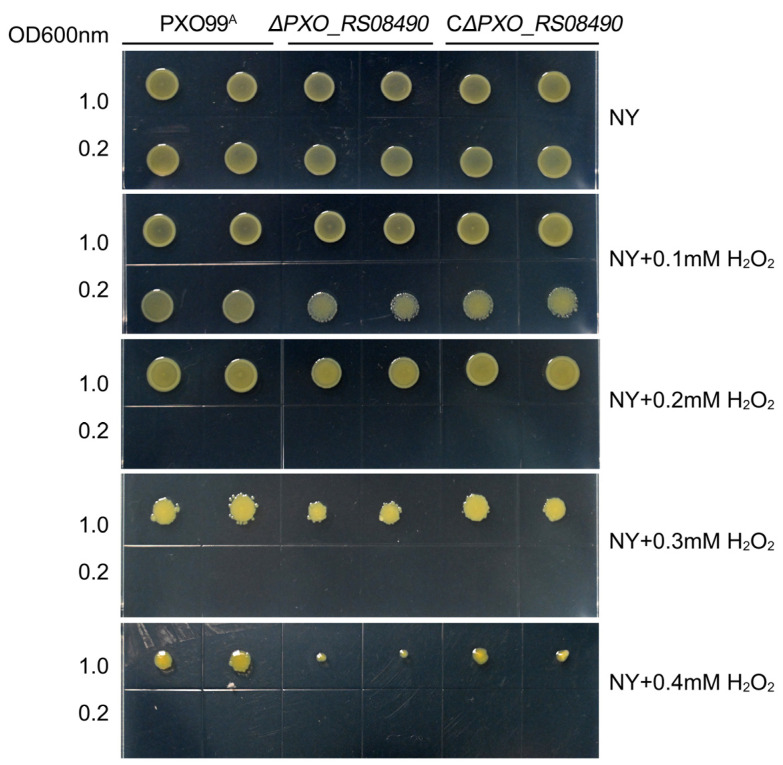
H_2_O_2_ resistance assay of PXO99^A^ and *PXO_RS08490* deletion mutants. Fresh bacterial suspensions were adjusted to an OD600 nm of approximately 1. Subsequently, 2 μL of each suspension was cultured on NY plates containing 0, 0.1, 0.2, 0.3, and 0.4 mM H_2_O_2_ concentrations. The NY plates were incubated at 28 °C and photographed after three days. Each experiment was repeated three times.

The findings demonstrated that at a final concentration of 0.1 and 0.2 mM H_2_O_2_, the OD600 value was 1, and both the WT and mutant strains could grow normally on NY plates. However, low-density bacteria could not grow at 0.2 mM H_2_O_2_. When hydrogen peroxide concentration was higher than 0.3 mM H_2_O_2_, both WT and mutant strains showed serious growth defects, and Δ*PXO_RS08490* strain was more obvious (Figure 9). The complementary strain of Δ*PXO_RS08490* (CΔ*PXO_RS08490*) repaired the defect, indicating that the gene was a key factor in the response to exogenous oxidative stress.

These findings indicate that a low concentration of H_2_O_2_ does not impair bacterial growth, but higher concentrations can be inhibitory, particularly when bacterial density is low, making them more susceptible to this external stress. The absence of *PXO_RS08490* significantly reduces the bacteria’s antioxidant capacity, highlighting its crucial role in PXO99^A^ strain resistance to exogenous oxidative stress and in maintaining redox homeostasis both within and outside the cell.

## 4. Discussion

Bacterial regulatory pathways are intricate and interconnected with each other, with sRNAs influencing various cellular activities through direct and indirect targets. The trans217 sRNA has been identified as a crucial factor in bacterial pathogenicity, but its specific regulatory pathways and target genes remain unclear. This study aims to map the complete regulatory network of trans217 and understand its role in virulence. To achieve this, transcriptome sequencing will be used to investigate specific gene regulatory networks, while comparative transcriptome analyses will examine the global regulatory networks of bacterial sRNAs.

*Xoo* WT strain PXO99^A^ and sRNA 217 deletion mutant were cultured under both nutrient-deficient and nutrient-rich conditions, followed by transcriptomic sequencing. The findings demonstrated that deletion of trans217 led to significant differential expression of 24 genes in the PSA medium, with 3 genes upregulated and 21 downregulated. In the XOM2 medium, 135 genes exhibited differential expression, with 52 upregulated and 83 downregulated. The differentially expressed genes included those encoding T3SS components, ABC transporters, oxidases, and glutamate synthases. Comparing the two culture conditions, the number of DEGs in the XOM2 group was much higher than that in the PSA culture condition (Figure 3, Figure 4, Figure 5 and Figure 6). XOM2 can be used as a plant-mimicking medium due to its simple composition and nutritional hunger. Especially when studying the functional molecules related to pathogen–host interaction, XOM2 may induce the expression of virulent-associated genes efficiently. Compared to the nutrient-rich PSA medium, the expression of pectate lyases gene *pelL* was specifically induced in the XOM2 medium by RT-qPCR experiments [36]. A research study showed that *X. oryzae* pv. *oryzae* growth in XOM2 media resulted in HrpX-dependent transcription of the *raxX* and *raxST* genes that control production of the RaxX sulfopeptide [37]. Suitable culture conditions are a prerequisite for interesting discoveries, and this study indicates that XOM2 is a good choice for demystifying functional molecules in pathogenic bacteria.

Notably, KEGG enrichment of DEGs in the XOM2 medium indicated that sRNA 217 was closely associated with the secretion system. Consistent with our previous findings, the absence of sRNA 217 resulted in the loss of function of the PthXo1 effector secreted by the bacterial T3SS [31]. These results suggest that future research on sRNA 217 should focus on identifying its targets and elucidating its regulatory mechanisms, particularly in relation to the T3SS.

Three different genes identified from the transcriptome data were knocked out to assess their function. The results showed that the SOD-coding gene *PXO_RS08490* contributed to bacterial virulence, with its deletion leading to reduced virulence in the host plant rice. Further H_2_O_2_ stress tests on plate cultures demonstrated that the *PXO_RS08490* gene is critical for bacterial resistance to oxidative stress and is essential for maintaining redox homeostasis. This suggests that sRNA trans217 also plays a role in regulating the function of the bacterial antioxidant enzyme system. Overall, the results provide an overview of the global regulatory network of sRNA trans217, highlighting its role in regulating multiple gene types and thereby collectively governing bacterial virulence.

Reports on the sequencing of sRNA transcriptomes in plant pathogenic bacteria are scarce. The function of most sRNA molecules remains unclear, with many studies focusing solely on their identification rather than their functional characterization. However, with increasing information about sRNA trans217, its regulatory function is becoming more evident. The next step involves conducting an in-depth analysis of the differentially expressed genes identified from the transcriptome data. This will be complemented by bioinformatics prediction methods to identify the direct targets of trans217 sRNA and to study their regulatory functions.

## 5. Conclusions

Our transcriptome data elucidated the comprehensive regulatory network of sRNA trans217 involved in bacterial virulence. This sRNA was found to regulate the bacterial secretion system, antioxidant enzyme system, ABC transporter system, and other critical functions. One of its downstream regulatory genes, *PXO_RS08490*, which encodes an SOD, is essential for bacterial pathogenicity in host rice and for the antioxidant defense mechanisms necessary to maintain normal physiological processes and virulence-associated functions. However, the study of the underlying sRNA requires identifying direct targets and elucidating the regulatory mechanism of sRNA targets. Based on the current results, the understanding of sRNA trans217 is still inadequate. Fortunately, our findings highlight the significant connection between sRNA trans217 and the bacterial secretion system, which will be a crucial focus for future target identification and mechanistic studies. Given the limited reports on functionally characterized sRNAs in plant pathogenic bacteria such as *Xanthomonas*, our systematic research strategy may provide valuable insights for sRNA exploration in other species.

## Figures and Tables

**Table 1:** RNA-Seq reads statistics and comparison with PXO99^A^ reference genome.

Samples	Q30 (%)	GC Content (%)	Total_Reads	Mapped_Reads	Gene Numbers
PSA_217_1	94.55	61.10	8,206,307	7,298,583 (89%)	4503
PSA_217_2	94.71	61.36	8,290,772	7,588,419 (92%)	4500
PSA_217_3	93.84	61.27	8,051,830	7,224,667 (90%)	4485
PSA_PXO99_1	94.55	60.65	7,812,714	6,333,425 (81%)	4485
PSA_PXO99_2	94.69	60.70	8,192,639	6,621,761 (81%)	4493
PSA_PXO99_3	94.64	60.61	7,992,108	6,561,197 (82%)	4487
XOM2_217_1	93.83	60.87	8,059,737	7,251,912 (90%)	4505
XOM2_217_2	93.79	60.89	8,199,489	7,352,156 (90%)	4504
XOM2_217_3	93.68	60.72	7,770,330	6,905,891 (89%)	4508
XOM2_PXO99_1	94.57	60.33	7,923,055	6,573,845 (83%)	4507
XOM2_PXO99_2	94.59	60.51	8,532,656	7,177,531 (84%)	4506
XOM2_PXO99_3	94.47	60.28	8,325,762	6,789,990 (82%)	4499

## Data Availability

Data is contained within the article or Supplementary Materials.

## Supplementary Materials

The following supporting information can be downloaded at: https://www.mdpi.com/article/10.3390/microorganisms12081684/s1, Table S1: Information on genes tested and primers used in this study.
