# Supplementary material for: Genome-Wide Transcriptome Analysis of a Virulent sRNA, Trans217, in Xanthomonas oryzae pv. oryzae (Xoo), the Causative Agent of Rice Bacterial Blight
Source: Microorganisms. 2024 Aug 16;12(8):1684. doi: 10.3390/microorganisms12081684 (PMC11357379; doi:10.3390/microorganisms12081684)
Supplement: Supplementary file 1 [file microorganisms-12-01684-s001.zip › microorganisms-3145473-supplementary.pdf]

Supplemental Data

Additional File 1: Table S1. Information on genes tested and primers used in this study.

Gene	Primers / subjects
PXO_RS08490	Upstream homologous arm:<br>F: 5'-GGCACCTTGATGTTGTTTGC-3'<br>R: 5'-TCTCCATCGTGGCTGTGTCG-3'<br>Downstream homologous arm:<br>F: 5'-CGACTACATCAAGGCGTGGT-3'<br>R: 5'-GATGGGTGGGATGAACAGGT-3' / deletion
PXO_RS00350	Upstream homologous arm:<br>F: 5'-CAAGGCAACGACAATGGCGC-3'<br>R: 5'-CAATCCGCAAATCCATCTGG-3'<br>Downstream homologous arm:<br>F: 5'-ACATCCGTCTGGTCGTTTAC-3'<br>R: 5'-AGGCTTCGGAGGTAAATCGC-3' / deletion
PXO_RS13155	Upstream homologous arm:<br>F: 5'-GCAAGACGAAGAGAGTGATC-3'<br>R: 5'-CGCAGCATCCACCAAGAAAT-3'<br>Downstream homologous arm:<br>F: 5'-ACACGATGGCAGGCACTTAT-3'<br>R: 5'-AGTTGGTCTTGCTCGGGATC-3' / deletion
PXO_RS05005	F: 5'-GCAACTTCACTGATCGCGAT-3'<br>R: 5'-TTATTGATCGCCAGCTTGCC-3' / RT-qPCR
PXO_RS00415	F: 5'-AATGCTGAGGAGGGTAAGGG-3'<br>R: 5'-CACTAAAGTCACCGCCGAAC-3' / RT-qPCR
PXO_RS19070	F: 5'-CCATGTGCCGATCTTCATCC-3'<br>R: 5'-TGATCGGGGCTGATGTAGTC-3' / RT-qPCR
PXO_RS16310	F: 5'-ATGTTCTGTTCGGAAGTGCG-3'<br>R: 5'-TGTTGAGAAAGTTGCCCACG-3' / RT-qPCR
PXO_RS15465	F: 5'-CAAAGCATTACCCGTAGCCG-3'<br>R: 5'-AGACTCTTCCTGCGGCTTG-3' / RT-qPCR
PXO_RS10055	F: 5'-TGTTTGCACAGCTTCGATCA-3'<br>R: 5'-TGACTCAATCGCTGGAAGGT-3' / RT-qPCR
PXO_RS16120	F: 5'-GATTCCGAACTGAACCCTGC-3'<br>R: 5'-ATGTCTGACTCGCGCATTTC-3' / RT-qPCR
PXO_RS16165	F: 5'-TTTTCCCCAACCATTCCCCT-3'<br>R: 5'-GGATCCATCGCATTGACGTC-3' / RT-qPCR
PXO_RS17315	F: 5'-CCTACGTGCTGGTCAATCAC-3'<br>R: 5'-GAGTGCATGAGTTCGTCGTC-3' / RT-qPCR
PXO_RS10060	F: 5'-CCTGAGCGGTTTTCTGATGG-3'<br>R: 5'-ATGTGGGTCGATCAGGTTGT-3' / RT-qPCR

PXO_RS26780	F: 5’-GATCCAGGCCCATTCACCA-3’<br>R: 5’-ACCTCGATCAATGTCTGCCA-3’ / RT-qPCR
PXO_RS00930	F: 5’-TTTGAAAACGGCAAGCAGGA-3’<br>R: 5’-TCCACCGATACTGCCGAAAT-3’ / RT-qPCR
PXO_RS09180	F: 5’-TGTATTACTCGGACCTGGGC-3’<br>R: 5’-GCGAGTACTGCAACACCATT-3’ / RT-qPCR
PXO_RS00350	F: 5’-ACTTGCGCTGTCTCTATCGA-3’<br>R: 5’-TCTGCACACCAAGATGTTCG-3’ / RT-qPCR
PXO_RS13155	F：5’-ATTTCTTGGTGGATGCTGCG-3’<br>R：5’-CACATTTGGCGGAACAGGAA-3’ / RT-qPCR
PXO_RS00340	F: 5’-CCTTGCTGCTCTGCCTAAAG-3’<br>R: 5’-CTGACCGAACTGCATGATGG-3’ / RT-qPCR
PXO_RS00335	F: 5’-AGACGGTCTGGAGGTTGATG-3’<br>R: 5’-TCTACGATCGACCTGTCTGC-3’ / RT-qPCR
PXO_RS00365	F: 5’-GATCATTGGGCTTGCCTACG-3’<br>R: 5’-CTGCTCGTCCTTGCTCATTC-3’ / RT-qPCR
PXO_RS18005	F: 5’-AGTTGCTACGGTTCGAGTCA-3’<br>R: 5’-AGGTATACATGGGAGACGCG-3’ / RT-qPCR
PXO_RS26750	F: 5’-TAAATGCATCGAAGGACCGC-3’<br>R: 5’-GCCTTCACAGCTCAGCAATT-3’ / RT-qPCR
